# Electroacupuncture-Induced Attenuation of Experimental Epilepsy: A Comparative Evaluation of Acupoints and Stimulation Parameters

**DOI:** 10.1155/2013/149612

**Published:** 2013-03-26

**Authors:** Xuezhi Kang, Xueyong Shen, Ying Xia

**Affiliations:** ^1^Shanghai Research Center for Acupuncture and Meridians, Shanghai 201203, China; ^2^Shanghai University of Traditional Chinese Medicine, Shanghai 201203, China; ^3^The University of Texas Medical School at Houston, Houston, TX 77030, USA; ^4^Yale University School of Medicine, New Haven, CT 06510, USA

## Abstract

The efficacy of electroacupuncture (EA) on epilepsy remains to be verified because of previous controversies that might be due to the complexity of the effects induced by different acupoints and stimulation approaches adopted. Therefore, we investigated the effects of EA on epilepsy to determine the specific acupoints and optimal stimulation parameters in this work. Experimental epilepsy was induced by injecting kainic acid to the lateral cerebral ventricle of adult male SD rats. EA with a low-frequency (10 Hz/1 mA) or high-frequency (100 Hz/1 mA) current was applied to the epileptic model for 30 minutes starting at 0.5 hour after the injection. Four pairs of acupoints were tested, that is, Shuigou (DU26) + Dazhui (DU14), Jinsuo (DU8) + Yaoqi (EXB9), Neiguan (PC6) + Quchi (LI11), and Fenglong (ST40) + Yongquan (KI1). We found that (1) low- or high-frequency EA at different acupoints reduced epileptic seizures (*P* < 0.05 versus the control) with an exception of low-frequency EA at Neiguan (PC6) and Quchi (LI11); (2) low-frequency EA induced a better effect at Fenglong (ST40) plus Yongquan (KI1) than that of the other acupoints (*P* < 0.05); (3) there is no significant difference in the effects of high-frequency EA at these acupoints; and (4) the high-frequency EA elicited a greater effect than that of low-frequency EA in all groups (*P* < 0.05), with an exception at Jinsuo (DU8) + Yaoqi (EXB9). The EA-induced attenuation appeared 1–1.5 hours after EA with no appreciable effect in the first hour after EA in either the EEG or the behavioral tests. We conclude that EA attenuation of epileptic seizures is dependent on the stimulation parameters and acupoints and that the delay in appearance of the EA effect could be a reflection of the time required by the EA signal to regulate neural function in the central nervous system.

## 1. Introduction 

Epilepsy, manifesting as recurrent seizures induced by abnormal electrical discharges in the brain, is a grave neurological disorder and incurs devastating effects on both patients and their families. There are numerous etiological factors implicated in epileptic seizures, including genetic abnormalities, hypoxic/ischemic injury, tumors, and trauma [1–5]. However, the currently available treatment strategies against epileptic seizures are limited and are associated with serious adverse effects [6–8]. In fact, the current treatment of patients with seizure disorders follows a *hit-and-trial* routine involving trial of various drugs and/or combinations to see works better for the individual. In more than one-third of these patients, no drug has proven to be effective. This includes a huge proportion given the worldwide prevalence of 50 million patients with seizure disorders. Although surgical treatment is successful in certain types of epilepsy, it is expensive, associated with side effects, and may not be easily amenable to all patients. The annual cost of epilepsy is estimated around $12.5 billion in the United States alone, stemming mostly from loss of productivity by those with intractable or poorly controlled epilepsy [9]. This underscores the need for an alternative and effective epilepsy treatment. Toward this goal, we have recently made novel observations on utilization of acupuncture in epilepsy treatment. 

In traditional Chinese medicine, acupuncture use has a long history in the treatment of epilepsy. Owing to its reproducibility and objectivity, electroacupuncture (EA) is increasingly being accepted and utilized in modern standards of care. In the past, some Chinese and Western investigators demonstrated the attenuation of chemically induced seizures in animals following EA [10–15]. Several scientific investigations published in the Chinese literature have documented a therapeutic effect of acupuncture/EA on epilepsy [16–20]. However, the efficacy of acupuncture/EA in epilepsy is still uncertain due to the limitations of the existing clinical studies and the flaws in their design. First of all, none of these clinical reports adequately describe the selection of their controls. Moreover, some clinical reports have failed to demonstrate a therapeutic effect of acupuncture/EA in the chronic intractable epilepsy [21–23]. It is very difficult to design a randomized, double-blinded placebo-controlled study in patients with epilepsy. Therefore, a systematic and well-controlled bench study is of utmost importance in order to validate the efficacy of acupuncture/EA before extrapolating its use in a clinical setting.

Multitude of factors can influence the efficacy of EA. Optimal parameters are essential including selection of appropriate regions for stimulation (acupoints), frequency of stimulation, and the method employed for stimulation. Thus, it is possible for these earlier reports to bear inconsistencies in their results owing to the differences in acupuncture parameters and acupoints used. No prior study has been conducted to systematically compare effects of low- and high-frequency EA at different acupoints on epileptic seizures. 

In the present work, we have attempted to tackle three fundamental issues: (1) to verify the efficacy of EA in attenuation of epileptic seizures; (2) to examine if low- and high-frequency EA had different effects on epilepsy; and (3) to determine if different acupoints with the same EA parameters lead to different EA effects.

## 2. Materials and Methods

### 2.1. Animals

All animal procedures were performed in accordance with the guidelines of the Animal Care and Use Committee of Shanghai Research Center for Acupuncture and Meridians. Adult male Sprague Dawley rats with a body weight of 180–220 g were purchased from Shanghai Experimental Animal Center of Chinese Academy of Sciences and were housed at 22–25°C. All the rats were allowed to drink and eat freely and had a controlled circadian rhythm set as 12-hour light/12-hour dark. The animals were randomly grouped into blank control (*n* = 4) and epilepsy (*n* = 63) groups. Furthermore, the epileptic animals were randomly subdivided to a group of epilepsy only (*n* = 10) and epilepsy plus EA groups (*n* = 53). 

### 2.2. Equipment and Materials/Reagents

Kainic Acid (KA) was purchased from Sigma Corporation (USA). All other chemicals and reagents came from China National Pharmaceutical Group Corporation. Equipments used in the study were as follows: stereotaxic apparatus (Model SR-6R, Narishige, Japan), electroacupuncture apparatus (Model G6805-2, Shanghai Medical Instrument High-tech Co., China), oscilloscope (Model XJ4210A; Shanghai Xinjian Instrument & Equipment Co., Ltd., China), bioelectric amplifier (ModelML132, AD Instruments Pty Ltd., Australia), and PowerLab 4/25 data acquisition system (Model ML845, AD Instruments Pty Ltd., Australia).

### 2.3. Induction of Epileptic Seizures

The rats were anesthetized with intraperitoneal injection of 10% chloral hydrate (0.4 g/kg body weight) and fixed on the stereotaxic instrument. The head epidermis and subgaleal were cut to expose the fonticulus anterior, fonticulus posterior, and the parietal bone. We adjusted and made the fonticulus anterior 1 mm higher than fonticulus posterior and recorded (1) anterior fontanelle sagittal level as  *R*_1_; (2) anterior fontanelle coronal level as  *P*_1_; and (3) the height of the anterior fontanelle as  *H*_1_.

For microinjection into the lateral cerebral ventricle, a hole of less than 0.5 mm diameter was drilled at  *R*_1_ +0.2 mm (0.2 mm posterior the anterior fontanelle) and  *P*_1_ +1.5 mm (1.5 mm from the midline) to penetrate the skull without damaging the meninges. The microinjector filled with KA solution (0.7 *μ*L, 1 *μ*g/*μ*L) was inserted into the lateral cerebral ventricle at  *H*_1_  −4.2 mm (at a depth of 4.2 mm beneath   *H*_1_). The KA solution was then injected into the lateral ventricle over 10 minutes at a uniform speed. After the end of injection, the micro-injector was left in place for 10 minutes and then gradually withdrawn.

In the control animals, KA solution was replaced with saline but followed the same injection procedure and into the same ventricle as in the KA animals.

### 2.4. Evaluation of Epileptic Activity

Every 30 minutes, we conducted a single-blinded behavioral assessment based on the Racine's 5-point scales [24] and recorded the scores at 8-time points in (i.e., 0.5, 1, 1.5, 2, 2.5, 3, 3.5, and 4 hours after the injection of KA). The assessment criteria utilized was shown in Table 1.

### 2.5. Electroencephalography

Two holes of less than 0.5 mm diameter were drilled in the rat skull at the areas corresponding to the right frontal cortex and right parietal cortex, respectively. Recording and reference electrodes were fixed in the holes using the dental cement and then connected to the PowerLab 4/25 data acquisition system for electroencephalography (EEG) signals. EEG parameters were set at sampling rate 2 k/s; range 50 mV; 100 Hz low-pass and the AC power filter. Since it is impractical to record EEG in the awake state due to unavoidable huge signal noise, we recorded EEG while the animals were under anesthesia, that is, from the beginning of the experiment to 10–15 minutes after EA when the animals were awake from anesthesia. 

### 2.6. Electroacupuncture

**Figure 1:**
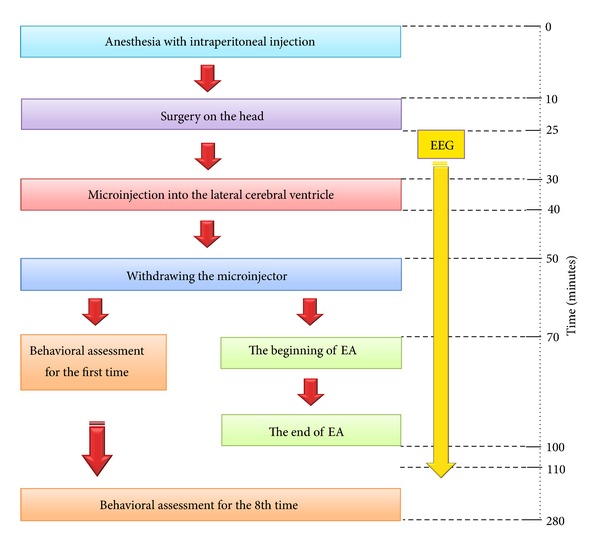
Experimental flow chart.

After the KA (epileptic model) or saline (control) injection into the lateral ventricle of the rat brain, the animals were randomly assigned to four major groups: head and neck acupoints (DU26, DU14), forelimb acupoints (PC6, LI11), the back acupoints (DU8, EXB9), and hindlimb acupoints (ST40, KI1). In a previous study, we had demonstrated that EA at an intensity of 1 mA is optimal for rats [25], thus in the present work we standardized this parameter and concentrated on the effects of different frequencies, which is another important determinant of the effect of EA [25]. In all the 4 groups, EA was given at both low- (10 Hz/1 mA) and high-frequency (100 Hz/1 mA). It was started 30 mins after the KA injection for a duration of 30 mins (see the experimental flow chart in Figure 1). 

### 2.7. Data Analysis

Data is presented as mean ± SE. Statistical significance was determined using either student's *t*-test or a one-way ANOVA. Statistical significance was defined by *P* < 0.05. All the data analysis and graphing were carried out using Origin 8.0 software (Origin Lab, USA).

## 3. Results

### 3.1. KA-Induced Seizures

**Figure 2:**
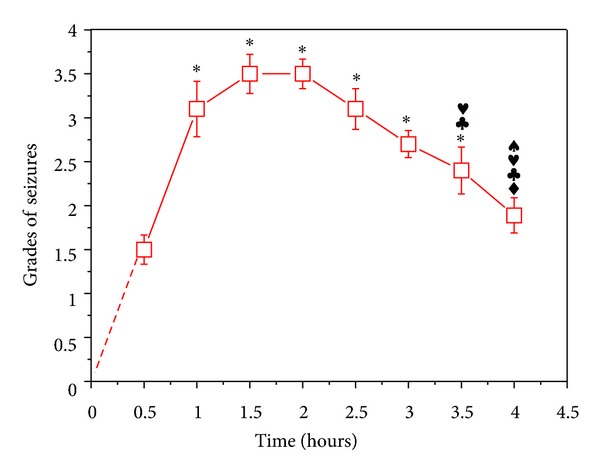
Change in grade of seizures against time after the KA injection. KA was injected in the lateral ventricle of the SD rats. *P < 0.05 versus that at 0.5 hour; ^♦^ *P* < 0.05 versus that at 1 hour; ^*♣*^ *P* < 0.05 versus that at 1.5 hours; ^*♥*^ *P* < 0.05 versus that at 2 hours; ^♠^ *P* < 0.05 versus that at 2.5 hours. Note that the onset of seizures appeared 0.5 hour after the KA injection, reached the peak 1.5–2 hours later, and then gradually started to decrease.

We injected 0.7 *μ*L of KA solution (1 *μ*g/*μ*L) into the lateral ventricle of each rat and applied Racine's 5-point scales grading method [24] to obtain the behavioral score. Ten to thirty minutes after the injection, the rats displayed seizure symptoms. The symptoms were most serious at 1.5–2 hours after the injection of KA and then gradually declined. However, epileptic seizure did not completely disappear within 4 hours after the injection (Figure 2). In the control group, same procedures were performed by replacing saline in place of KA, and they did not induce any seizures or any abnormal behaviors. 

### 3.2. KA-Induced EEG Changes

**Figure 3:**
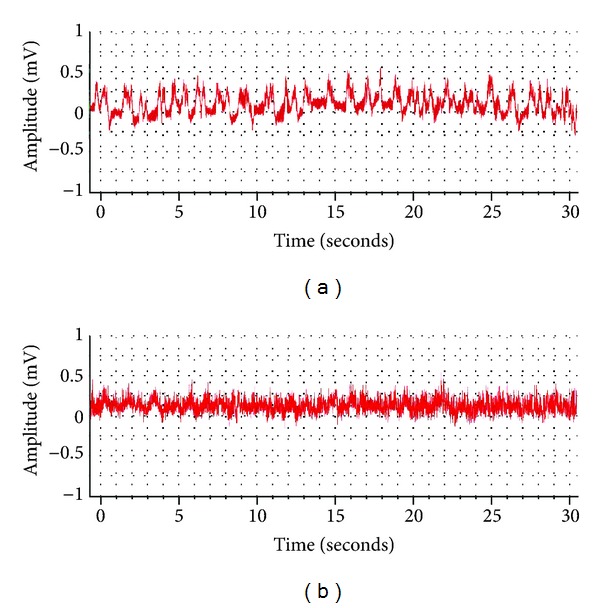
Representative EEG recordings from the rat before and after the KA injection. (a) Before KA injection and (b) 30 minutes after the injection. Note that KA induced an epileptic spike wave pattern with a large increase in frequency.

**Figure 4:**
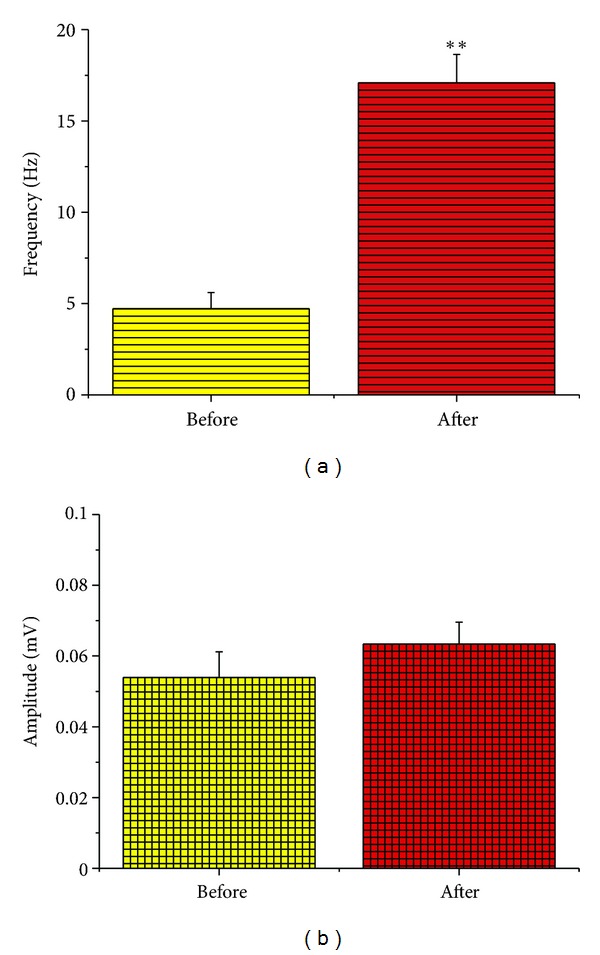
Quantitative changes in frequency and amplitude of EEG after the KA injection, (a) frequency and (b) amplitude. **P < 0.001. Note that simultaneous to the induction of seizures, the frequency of the EEG waves increased more than 3.5 folds. In contrast, the amplitude had no significant change (*P* > 0.05).

The epilepsy-like spike wave-pattern could be clearly seen on EEG after KA injection (Figure 3). Frequency on EEG wave pattern increased significantly after KA injection (from 4.75954 ± 0.90465 Hz to 17.26694 ± 1.57080 Hz, *n* = 51, *P* < 0.001) (Figures 3(a) and 4(a)). Although there was an increase in the amplitude of the EEG waves in the KA model, it had no statistical difference when compared to the control group, that is, before the KA injection (from 0.05395 ± 0.00722 mV to 0.06342 ± 0.00612 mV, *n* = 51, *P* > 0.05; Figure 4(b)).

### 3.3. Effects of Low- and High-Frequency EA at Different Acupoints

**Figure 5:**
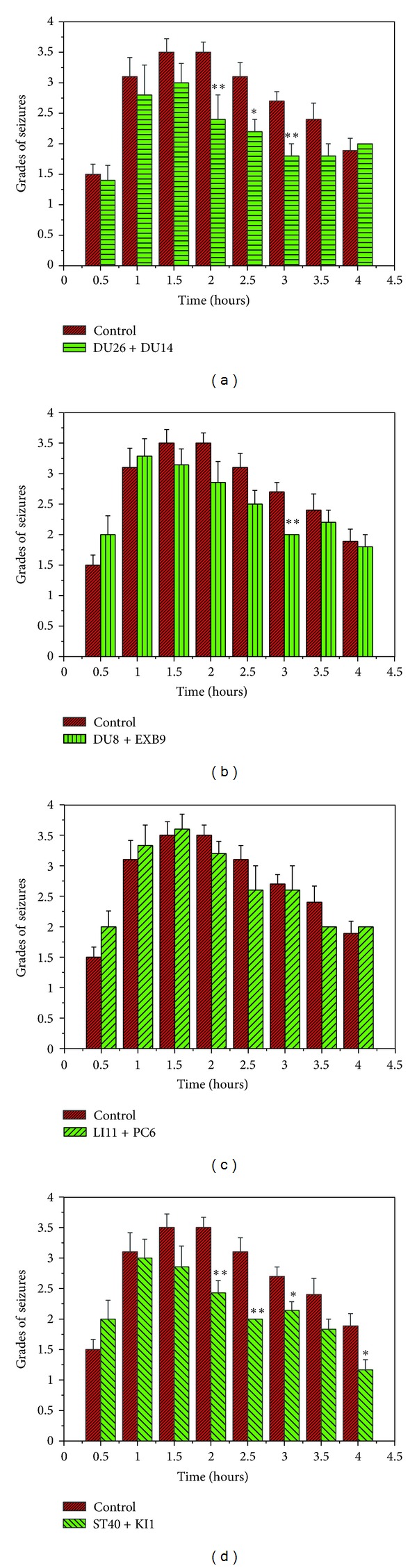
Effects of 10 Hz/1 mA EA at different acupoints. (a) DU26 + DU14 (*n* = 5); (b) DU8 + EXB9 (*n* = 7); (c) LI11 + PC6 (*n* = 6); and (d) ST40 + KI1 (*n* = 7). *P < 0.05, **P < 0.01 EA groups versus the control. Note that EA at the acupoints of the head and neck (DU26 + DU14), the back (DU8 + EXB9), and the hindlimb (ST40 + KI1), but not the forelimb (LI11 + PC6), significantly attenuated seizures at several points after the KA injection.

**Figure 6:**
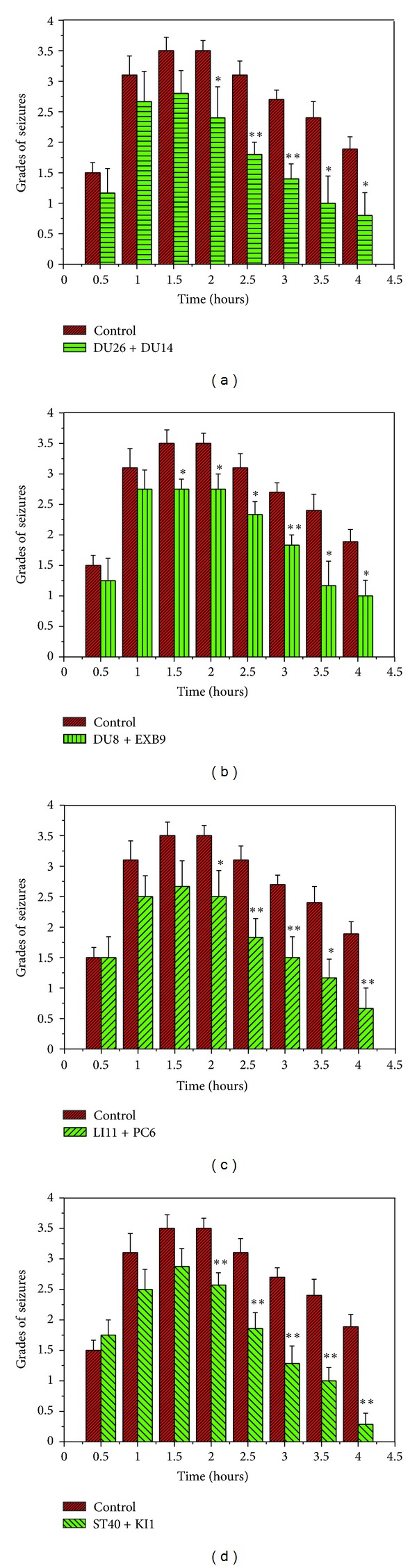
Effects of 100 Hz/1 mA EA at different acupoints. (a) DU26 + DU14 (*n* = 6); (b) DU8 + EXB9 (*n* = 8); (c) LI11 + PC6 (*n* = 6); and (d) ST40 + KI1 (*n* = 8). *P < 0.05, **P < 0.01 EA groups versus the control. Note that EA with high frequency stimulation (100 Hz/1 mA) at all these acupoints induced a significant reduction in seizures at most time points, 1–1.5 hours following the KA injection.

**Figure 7:**
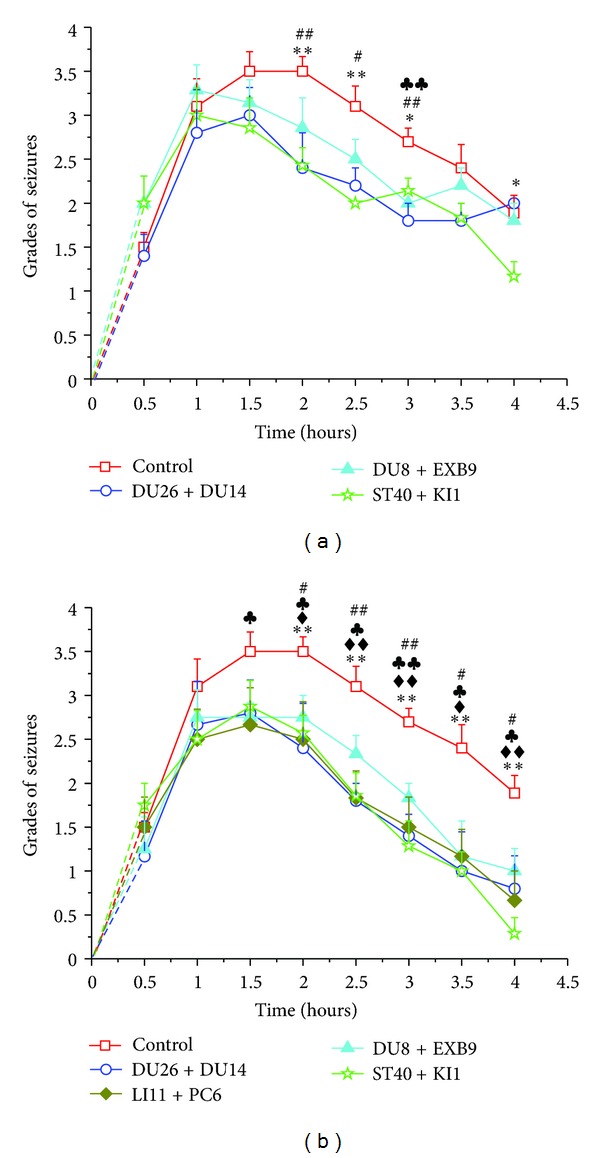
Comparisons of EA effects among “effective” acupoints. (a) 10 Hz/1 mA and (b) 100 Hz/1 mA. ^#^ *P* < 0.05, ^##^ *P* < 0.01 DU26 + DU14 versus control; ^*♣*^ *P* < 0.05, ^*♣♣*^ *P* < 0.01 DU8 + EXB9 versus control; ^♦^ *P* < 0.05, ^♦♦^ *P* < 0.01 LI11 + PC6 versus control; and *P < 0.05, **P < 0.01 ST40 + KI1 versus control. Note that there is no statistical difference among the different “effective” acupoint groups.

We utilized two EA frequencies, that is, 10 Hz/1 mA and 100 Hz/1 mA, that have been previously described in the acupuncture literature [25–30], to compare the role of different acupoints in EA effect (Figures 5–7). We tested the following acupoints: DU26 and DU14 in the head and neck, PC6 and LI11 in the forelimb, DU8 and EXB9 in the back, and ST40 and KI1 in the hind limb and found that both EA frequencies were effective in reducing seizures (Figures 5 and 6). 

As shown in Figure 5, the low-frequency stimulation (10 Hz/1 mA) at the head and neck, back, and the hind limb acupoints significantly attenuated seizures at several points in time after KA injection. However, EA at PC6 and LI11 did not induce any significant reduction in seizures during the period investigated (Figure 5(c)). 

On the other hand, high-frequency stimulation (100 Hz/1 mA) at all the aforementioned acupoints induced a significant reduction in seizures 1–1.5 hours following the KA injection (Figure 6). 

With the same stimulating parameters, the comparison among different “effective” acupoints did not show any significant difference in terms of EA-induced effect on the KA-induced seizures (Figure 7).

### 3.4. Difference in Low- versus High-Frequency EA on the KA-Induced Seizures

**Figure 8:**
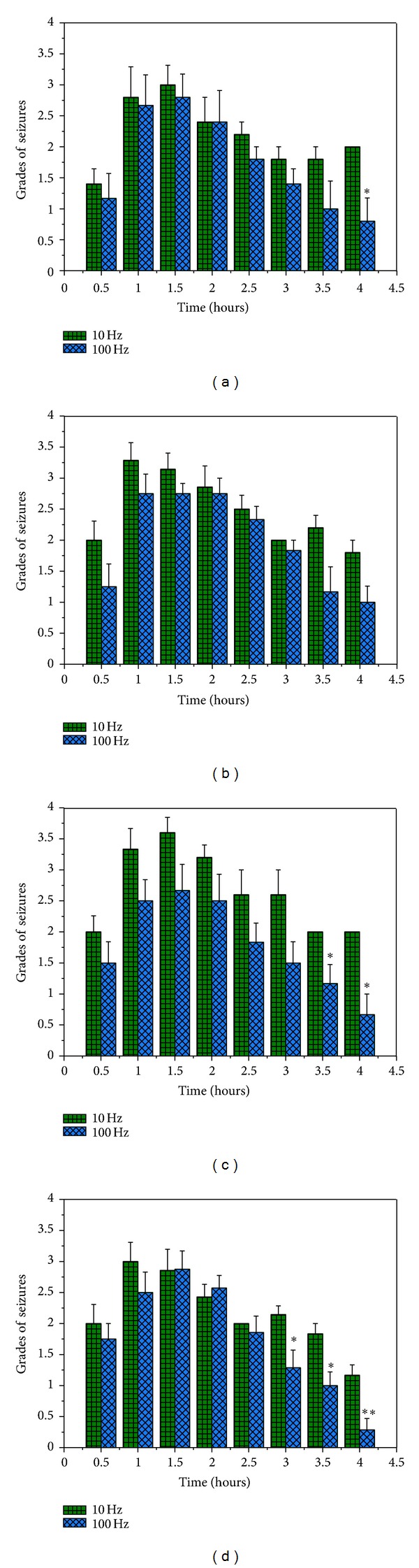
Comparisons of low- and high-frequency EA effects on the seizures. Discrepant seizure levels in four acupoint groups after EA with the parameters of 100 Hz/1 mA or 10 Hz/1 mA. (a) DU26 + DU14; (b) DU8 + EXB9; (c) LI11 + PC6; and (d) ST40 + KI1. *P < 0.05 or **P < 0.01 versus 10 Hz. Note that except for the group of DU8 + EXB9, all other three groups showed a greater EA effect with 100 Hz/1 mA than 10 Hz/1 mA.

When comparing 100 Hz/1 mA to 10 Hz/1 mA, we found that the high-frequency EA induced a greater attenuation of seizures. As shown in Figure 8, except DU8 + EXB9, all other groups achieved a larger reduction in seizure activity after high-frequency EA when compared to low-frequency EA. 

### 3.5. Effect of EA on EEG

We examined the changes in EEG frequency and amplitude from the initiation of EA (30 minutes after the KA injection) to 10 minutes after stopping EA (70 minutes after the KA injection). No significant changes in the frequency and amplitude were noted either in low- or high-frequency EA groups, or in “effective” versus ineffective acupoint groups. The results are summarized in Tables 2 and 3. 

## 4. Discussion

This is the first study conducting a systematic comparison of different EA stimulation parameters and acupoints in terms of their effects on epileptic seizures. Our results show that (1) EA significantly attenuates KA-induced epilepsy; (2) EA-induced attenuation appears 1–1.5 hours after EA with no appreciable effect from the start of application to 1 hour after EA; and (3) the EA effect was relatively specific to acupoints and EA parameters.

There has been a longstanding controversy in the published literature on the clinical efficacy of EA treatment in epilepsy. Because of the complex etiology and presentation of epilepsy, there are a multitude of factors that can influence the EA effect. Furthermore, it is not feasible to make comparisons as the data were obtained from a varied pool of patients and animal models that utilized different acupuncture/EA parameters and acupoints. Our present study employs a commonly used rat model of epilepsy and provides strong evidence on the attenuation of certain types of epileptic seizures following EA treatment, such as chemical-induced seizures (e.g., KA-induced epilepsy).

It is noteworthy that we failed to observe any significant reduction in epileptic seizures during or immediately after the EA treatment, neither on EEG nor on behavioral assessments. Instead, we found a delay in attenuation of epilepsy following EA that appeared 1 hour after stimulation. Our unique observations stand in contrast to some of the previous studies [26, 27, 29–31], in which the investigators stated that acupuncture/EA induces an immediate inhibition of epileptic activity [27, 31]. At present, we cannot explain the reasons for such a contrasting effect. However, we believe that this disparity can be attributed to the difference in the methods of EA stimulation in the epilepsy models employed by us and the others. However, we found a few reports documenting a rapid effect of EA using a similar KA-induced epilepsy model of as ours [26]. 

Among the four major acupoint groups tested, we found that low-frequency (10 Hz/1 mA) EA at head and neck acupoints (DU26 + DU14), back acupoints (DU8 + EXB9), and hindlimb acupoints (ST40 + KI1) induced a significant attenuation of epileptic seizures, while stimulation of forelimb acupoints (PC6 + LI11) had no such effect. These results suggest that the EA effect is relatively specific to acupoints. This could be due to the difference in neural distribution around the acupoints [32, 33] because of differences and complexity in neural transmission from various acupoints to the brain and spinal cord [32–36].

Interestingly, the effectiveness of acupoints varies under different stimulating parameters. For example, the low-frequency EA at the forelimb acupoints (PC6 + LI11) was not effective for epileptic seizures, but the high-frequency EA at the same acupoints induced a significant reduction in epileptic seizures. In general, the high-frequency EA produced a better effect compared to that of low-frequency EA at all acupoints. Based on these observations, we consider high-frequency stimulation as “an optimal parameter” for EA treatment of the KA-induced epilepsy. The underlying mechanism may be related to specific changes in neural signals generated in the body. High-frequency stimulation may produce neural signals to affect broader brain regions, of which some may respond only to specific acupoints in the case of low-frequency currents. 

Several lines of evidence suggest that *δ*-opioid receptors (DORs) mediated regulation of sodium channels may play a significant role in the molecular mechanism of the EA-induced attenuation of epilepsy [1]. It is well known that neural hyperexcitability during the epileptic seizures may result from dysregulation of sodium channels in the brain [1, 37]. In a mutant model of spontaneous epilepsy [37, 38], for example, sodium channel expression was found to be high with increased sodium currents and hyperexcitability in the cortical regions [37]. A concomitant low DOR expression was also found in the same model [39]. Furthermore, we found that DOR expression and/or activation decreases sodium currents [40]. Extrapolating from these observations, we hypothesize that DOR expression/activity decreases the sodium channel function thereby reducing sodium currents and hyperexcitability. An impairment of DOR function, on the other hand, will lead to an increase in sodium currents and hyperexcitability that in turn results in an epileptic seizure. Since acupuncture/EA is known to upregulate endogenous opioid expression and function in the brain [34, 36] and increase DOR expression in the cortex [41], we believe that EA upregulates DOR expression and/or function, thus inhibiting epileptic seizures mediated by neural hyperexcitability due to sodium channel dysfunction. This may also partially explain that EA induced a delayed, not immediate, effect on epilepsy because the upregulation of DOR, particularly expression, requires a certain period of time to make the action in the brain.

In summary, our study reveals that EA induced a significant reduction of epileptic seizures with high frequency as an optimal parameter for EA efficacy in the KA-induced epilepsy model. The EA effect is a delayed response, which is likely to be related to the time involved in the neural regulation of central functions. However, there is a caveat attached that the optimal acupuncture parameters and the underlying mechanisms may vary with different types of epilepsy due to diverse pathophysiological factors causing epileptic seizures. Further investigations on the optimal parameters and mechanisms involved in EA treatment of epilepsy are needed to improve our current knowledge and shed light on better solutions of this neurological disorder. 

## Figures and Tables

**Table 1:** Racine's 5-point scales*.

Grades	Behavioral changes
1	Mouth and facial movement
2	Head nodding
3	Forelimb clonus
4	Rearing with forelimb clonus
5	Rearing and falling with forelimb clonus (generalized motor convulsions)

*Score 0: without any abnormal activity/sign (i.e., the status before the injection KA).

**Table 2:** Frequency & Amplitude of EEG before and after 10 Hz/1 mA EA.

Acupoints	Frequency (Hz)		Amplitude (*μ*V)	
	Before EA	After EA	Before EA	After EA
DU26 + DU14 (*n* = 5)	24.14 ± 9.54	14.44 ± 11.32	44.04 ± 47.73	56.28 ± 47.16
DU8 + EXB9 (*n* = 7)	13.89 ± 11.04	21.27 ± 12.00	55.47 ± 41.97	53.16 ± 51.02
LI11 + PC6 (*n* = 6)	23.61 ± 12.08	19.27 ± 13.85	39.27 ± 30.14	26.92 ± 35.52
ST40 + KI1 (*n* = 7)	22.41 ± 12.00	18.37 ± 14.87	44.17 ± 39.18	22.16 ± 21.11

Before EA: Before the initiation of EA (30 minutes after the KA injection).

After EA: 10 minutes after the end of EA (70 minutes after the KA injection).

**Table 3:** Frequency & Amplitude of EEG before and after 100 Hz/1 mA EA.

Acupoints	Frequency (Hz)		Amplitude (*μ*V)	
	Before EA	After EA	Before EA	After EA
DU26 + DU14 (*n* = 6)	13.57 ± 12.35	12.85 ± 12.82	110.48 ± 72.37	122.10 ± 160.36
DU8 + EXB9 (*n* = 7)	13.88 ± 13.03	21.29 ± 14.20	64.40 ± 23.86	52.10 ± 39.08
LI11 + PC6 (*n* = 6)	15.28 ± 9.44	16.57 ± 17.27	74.33 ± 35.56	63.42 ± 37.38
ST40 + KI1 (*n* = 7)	13.41 ± 7.56	15.33 ± 11.01	74.46 ± 21.65	65.58 ± 54.1

Before EA: Before the initiation of EA (30 minutes after the KA injection).

After EA: 10 minutes after the end of EA (70 minutes after the KA injection).
